# Renal excretion of 1,2-dihydroxynaphthalene (DHN) in firefighting instructors after exposure to polycyclic aromatic hydrocarbons (PAHs) during live fire training

**DOI:** 10.1038/s41598-024-62388-2

**Published:** 2024-07-02

**Authors:** Felix Lang, Daniel Wollschläger, Dipl.-Ing. Stephan Letzel, Bernd Roßbach

**Affiliations:** 1 Institute of Occupational, Social and Environmental Medicine, University Medical Center, Johannes Gutenberg-University, Obere Zahlbacher Strasse 67, 55131 Mainz, Germany; 2 Institute of Medical Biostatistics, Epidemiology and Informatics (IMBEI), University Medical Center, Johannes Gutenberg-University, Mainz, Germany

**Keywords:** Biomonitoring, Naphthalene, Kinetics, Urine, Half-life, Diagnostic markers, Occupational health

## Abstract

Exposure of firefighting instructors to polycyclic aromatic hydrocarbons (PAHs) such as naphthalene is unavoidable during live fire training. The study aimed to investigate naphthalene uptake by measuring the urinary excretion of the naphthalene metabolite 1,2-dihydroxynaphthalene (DHN), to describe the DHN elimination kinetics and to evaluate the results by comparison to further biomarkers of PAH exposure. N = 6 male non-smoking firefighting instructors completed five training sessions each in a residential fire simulation unit under respiratory protection. All participants provided two urine samples before and another seven samples within an 18-h-interval after each session. DHN was detected by gas chromatography/tandem mass spectrometry (GC–MS/MS) in all samples (n = 237) with median concentrations ranging from 3.3 µg/g crea. (range 0.9–10.2) before exposure to 134.2 µg/g crea. (43.4–380.4) post exposure. Maximum elimination found 3.3 h (median) after onset of exposure decreased with a mean half-life of 6.6 h to 27.1 µg/g crea. (15.7–139.5) 18 h after training. DHN sensitively indicated a presumed dermal naphthalene intake during training, showing similar elimination kinetics like other naphthalene metabolites. Internal exposure of the participants transiently exceeded exposures determined for non-smokers in the general population, but was lower than at other workplaces with PAH exposure. Despite limited uptake, accumulation is possible with daily exposure.

## Introduction

Flashovers as a widespread sudden ignition of combustible material in an enclosed area are a major cause of traumatic injury to firefighters^1,2^. Flashovers are often preceded by smoke ignition (rollover)^3,4^. Controlled rollover scenarios are created in live fire systems for training purposes to prepare firefighters for these hazards. In such live fire situations, firefighters are inevitably exposed to combustion products such as PAHs with repeated exposure in particular for firefighting instructors^5–7^. Despite the use of protective clothing, self-contained breathing apparatuses (SCBAs) and hygiene measures, the uptake of PAHs by instructors during live fire training is well documented^6–11^.

Given adequate respiratory protection, dermal absorption of PAHs is considered a relevant route in this context as studies show that PAH-contaminated air can penetrate protective clothing to the skin surface^11,12^, reviewed in^13^. In particular, low molecular weight compounds such as naphthalene (NAP) are found in the air under protective clothing. This is partly due to the higher concentrations of these compounds in fire emissions compared to more complex PAHs such as pyrene (PYR). On the other hand, the protective effect of clothing appears to be lower for volatile (vaporous) PAHs^11,12,14^. In addition, higher dermal absorption rates seem to facilitate uptake of low molecular weight PAHs as it is as known from in vivo and in vitro experiments [reviewed in^13^]. From the toxicological point of view, low-molecular-weight PAHs are suspected to elicit co-cancerogenic effects during concurrent exposure to genotoxic PAHs^15,16^. NAP itself is currently categorised in group 2B (possibly carcinogenic to humans) by IARC^17^. For these reasons, it is worth considering also low-molecular-weight PAHs, especially NAP, when assessing firefighters' PAH exposure.

However, data on naphthalene skin contaminations in fire fighters is scarce, which may also be due to a considerable volatility of the substance hampering a reliable quantification of post exposure levels on the skin surface^9,10,18^. Instead, human biomonitoring is advisable for exposure assessment^11,19^. Urinary PAH metabolites such as 1-hydroxypyrene (1-PYR) or 1- and 2-hydroxynaphthalene (1-NAP, 2-NAP, “naphthols”) have been commonly used to assess internal exposure of firefighters by human biomonitoring^20^. While excretion of the pyrene metabolite 1-PYR is traditionally measured as a biomarker of PAH exposure already since the mid-80 s of the last century^21^, naphthalene metabolites are of particular interest due to the aforementioned considerations on naphthalene abundance and its potential for dermal exposure and uptake. Metabolites such as 1-PYR or 1- and 2-NAP are formed by cytochrome P-450 epoxidation of the parent compounds, followed by spontaneous rearrangement to yield the corresponding phenols. After conjugation with glucuronic acid or sulfate, the latter can be eliminated from the body by renal excretion. Alternatively, aromatic dihydroxy compounds such as 1,2-dihydroxynaphthalene (DHN) can be formed from the primary epoxides by epoxide hydrolase mediated hydrolysis and subsequent oxidation catalysed e.g. by aldo–keto-reductases. Like phenols, also dihydroxymetabolites are excreted in urine after conjugation^22,23^. With respect to naphthalene, 1,2-dihydroxynaphthalene (DHN) has been proposed as a relevant urinary biomarker since it is excreted in even higher amounts than 1- or 2-NAP^24,25^. As main naphthalene metabolite, DHN indicated inhalation exposure more sensitively and showed closer correlation with external exposure than both naphthol biomarkers^25^. Despite these advantages, the parameter has only been used sporadically due to the unavailability of suitable analytical standards and the instability of (unconjugated) 1,2-DHN in aqueous solutions. Furthermore, there is a lack of information on the renal excretion kinetics of DHN in humans, especially after dermal absorption^20,25,26^.

The aim of the study was to investigate the uptake of naphthalene during live fire training in a group of firefighting instructors using DHN in urine as marker for internal exposure and to compare these results to data for the already established biomarkers 1-, 2-NAP and 1-PYR. Furthermore, the excretion kinetics of DHN should be explored. This article presents additional data and supplementary evaluations to the previously published results of the study by Rossbach et al.^6^.

## Methods

### Subjects

All study participants (n = 6) were male firefighting instructors from Germany. The instructors were aged between 25 and 41 years (median 35 years). None of the participants wore a beard, which could potentially affect the seal of the SCBA face piece. Before the start of the study, all subjects declared that they were non-smokers. Tattoo bearers were excluded before the start of the study as tattoo ink can be a potential source of polycyclic aromatic hydrocarbons^27^. The authors declare that this research was conducted in accordance with the relevant regulations, including the Declaration of Helsinki. All subjects gave written informed consent after being informed about the study by the investigator. The study protocol was approved by the Ethics Committee of the Medical Association of Rhineland-Palatinate, Germany, before the start of the study (reference number 2018-13952 - Clinical Research).

### Training facilities and procedure

Exposure to combustion products took place in several training sessions that simulated live fire conditions by burning a defined number of uncoated chipboards in an overseas container. The container was located in a hall with exhaust air purification. The participants entered the training hall wearing full personal protective equipment consisting of a firefighting suit, helmet, flame-resistant hood, gloves, boots and self-contained breathing apparatus (SCBA) in accordance with European standards (e.g. EN 469). In addition, all subjects wore long-sleeved undergarments under their turnout gear. Only laundered clothing and cleaned equipment (e.g. helmets, masks) were used. The donning and doffing of clothing and protective equipment took place only in a smoke-free, well-ventilated area. Immediately after taking off the firefighters' gloves after training, the participants put on nitrile gloves for further undressing. After removing the helmet, flash hood and SCBA face shield, the participants put on particle filtering half masks (FFP3) to remove contaminated turnout jackets and trousers. After a break for rehydration and cool down still wearing their undergarments, the participants went to shower. The total duration of a training session was 1.5–2.0 h (mean 1.75 h), including donning and doffing of protective equipment, time spent in the fire training facility (about 30–45 min), and final body cleaning. The interval between two consecutive training sessions for each subject was at least six days. All subjects collected urine in separate collection vessels at nine predefined times (Table 1): in the morning of each exposure day (sampling 1), immediately before each training session (sampling 2), immediately after each training session (sampling 3) as well as 1, 3, 6, 9, 11 and 18 h post exposure (samplings 4 to 9). In this way, each subject was biomonitored at each of the five training sessions. All additional (unscheduled) urinations between the predefined times were also collected in separate containers and pooled afterwards with the next scheduled sample. Like this, a set of nine samples representing the total urine output over 24 h was obtained for each participant and each training session. A total of 270 samples were to be collected using this procedure for all participants and five training sessions of each individual. Subjects completed a questionnaire for each sampling period to account for possible pre-exposure or concurrent exposure to PAHs from sources other than the fire simulation unit, such as occupational exposure to combustion products, exposure to tobacco smoke (active/passive) and barbecue activities, and consumption of smoked or charbroiled foods.

**Table 1:** Schedule for urine sampling (samplings 1 to 9) in chronological relation to the training session.

Sampling	Default sampling time	Mean time start exposure—sampling (range) (h)
1	Morning urine after getting up on training day (approx. 06.00–07.00 h)	− 4.2 (− 5.3 to − 2.3)
2	Before training	− 0.4 (− 1.0 to − 0.1)
3	At the end of the training session (after removing protective clothing and cleaning the body)	2.0 (1.5 to 2.4)
4	1 h after the end of training	3.0 (2.5 to 3.6)
5	3 h hours after the end of training	5.2 (4.5 to 5.6)
6	6 h after the end of training	8.1 (7.4 to 8.6)
7	9 h after the end of training	11.2 (9.6 to 12.8)
8	right before bedtime (approx. 22.00–24.00 h)	12.7 (11.6 to 14.1)
9	Morning urine after getting up the next day (approx. 06.00–07.00 h)	20.1 (18.2 to 21.8)

All subjects collected urine in separate collection vessels at nine predefined times. Additional (unscheduled) urinations between the predefined times were also collected in separate containers and pooled afterwards with the next scheduled sample. Like this, a set of nine samples representing the total urine output over 24 h was obtained for each participant and training session.

### Analytical determinations

The mono-hydroxylated PAH metabolites 1-NAP, 2-NAP and 1-PYR in urine were determined as described elsewhere^6^ referring to a procedure of^28^. In brief, the analytes were extracted by liquid–liquid extraction with n-hexane after enzymatic hydrolysis of the urine samples with ß-glucuronidase/arylsulfatase (isolated from helix pomatia) and derivatised by silylation with N-trimethylsilyl-N-methyl trifluoroacetamide afterwards. Gas chromatography-tandem mass spectrometry (GC–MS/MS, Agilent 5975C/Evolution^3^, Chromtech GmbH, Bad Camberg, Germany) was used to analyse the pretreated samples achieving detection limits of 0.1 µg/l (1- and 2-NAP) and 0.02 µg/l (1-PYR), respectively. External calibration with internal standardisation by isotopically labelled analogs of the target compounds (D8-1-NAP, D7-2-NAP, 13C6-1-PYR) was done by analysis of pool urine samples spiked with the unconjugated metabolites. All standards were obtained from LGC-Standards, Wesel, Germany. Repeated analyses of control samples prepared in pool urine (n = 10, spiking concentration 0.34–0.65 µg/l) in parallel with the study samples resulted in between day imprecisions between 12.8 and 15.6%. Accuracy of the results was confirmed by successful participation in the German External Quality Assessment Scheme for analyses in biological material (G-EQAUS, www.g-equas.de).

DHN in urine was determined referring to a method described in detail by Klotz et al.^29^. After enzymatic hydrolysis with ß-glucuronidase/arylsulfatase (isolated from helix pomatia), sample clean-up by solid-phase extraction (Bond Elut PPL 100 mg/3 ml, Agilent Technologies Deutschland GmbH, Waldbronn, Germany) and silylation with bis(trimethylsilyl)acetamide/trimethylchlorosilane, the trimethylsilylderivative of DHN was separated from the sample matrix by gaschromatography and detected by tandem mass spectrometry (Agilent 5975C/Evolution^3^, Chromtech GmbH, Bad Camberg, Germany) with a detection limit of 0.3 µg/l. External calibration with internal standardisation by an isotopically labelled analogue of the target compound was done by analysis of pool urine samples spiked with the glucuronides of DHN and D6-1,2-dihydroxynaphthalene (internal standard) before enzymatic hydrolysis. Both standards are not commercially available and were obtained as a gift from the Institute of Occupational, Social and Environmental Medicine at the University of Erlangen-Nuremberg (Germany). A detailed description of the synthesis of the compounds is given by^26^. Repeated analyses of control samples prepared in pool urine (n = 6, spiking concentration between 7.0 and 502 µg/l) in parallel with the study samples resulted in between day imprecisions below 9.4%. Up to now, there is no external quality assessment scheme available for DHN.

Urinary creatinine was analysed in the Institute of Clinical Chemistry and Laboratory Medicine of University Medical Centre of the Johannes Gutenberg University Mainz using a photometrical assay based on the method of^30^. Only samples with a creatinine content between 0.3 and 3.0 g/L were included in the statistical analyses, following a recommendation of the World Health Organization^31^. Urinary metabolite concentrations are given in relation to creatinine to further minimise dilution effects by hypo- and hyperhydration of the study participants.

### Statistics

Descriptive analysis was conducted using SPSS 29 (IBM Deutschland GmbH, Ehningen). Metabolite concentrations below the LOD were substituted by a value equal to ½ of the respective LOD. Percentiles (25th, 50th, 75th) were calculated for statistical data description. Relative changes in metabolite concentrations between two samplings (e.g. sampling 2 and 4) were calculated by dividing the concentration of both samplings (c4)/c2) for a particular individual and training session. Aggregated relative changes are reported as median values. Pearson correlation coefficients (r) were calculated to analyse for correlations between the logarithmic concentrations of the individual metabolites across all samplings.

### Initial half-life

The initial half-life of each biomarker was estimated using statistical methods described previously^6,32^. In short, we estimated the first-order elimination rate constant λ based on the model in Eq. (1) ^32^:

1 $$ {\text{C }} = {\text{C}}_{0} + {\text{B}} \cdot {\text{ e}}^{{\lambda \cdot {\text{t}}}} + \in $$

Here, C is the creatinine-adjusted concentration in urine after the uptake phase, C_0_ is the baseline concentration before training, B is the difference from peak to baseline concentration, t is the time since peak concentration, and ϵ is the error term. Only half-lives for a first excretion phase could be estimated since the follow-up did not last until pre-exposure concentrations were reached again. Using package nlme^33^ for the statistical environment R^34,35^, the model was fitted as a linear mixed effects model for the log concentration difference to baseline ln(C–C_0_) as the outcome. The model was fitted simultaneously for all data and included fixed effects for biomarker, time since peak, and their interaction term. It used random slopes for time since peak and random intercepts for participant nested within training session. Errors were assumed to be autocorrelated according to an AR(1) process.

## Results

Each of the six participants completed five training sessions resulting in 30 individual sample sets. Of the total of 270 samples expected from nine consecutive samplings per sample set, 268 could be analysed. One sample was accidentally discarded and one scheduled sample was not collected. 237 samples met the inclusion criterion of a creatinine level between 0.3 and 3.0 g/l for further analysis. While DHN and 2-NAP were detected in all samples, 1-NAP and 1-PYR were found in 96% of the samples, each.

Table 2 provides an overview over the creatinine normalised DHN concentrations together with the concentrations for 1-NAP, 2-NAP and 1-PYR, stratified by sampling time. Before training (sampling 1 and 2) we measured median baseline concentrations of 2.23 and 3.32 µg/g crea. for DHN (total range: 0.87–10.19). While the corresponding concentrations for 2-NAP (2.00 and 1.94 µg/g crea.) were comparable to DHN, the pre exposure concentrations for 1-NAP (0.61 and 0.39 µg/g crea.) and 1-PYR (0.06 and 0.05 µg/g crea.) were considerably lower.

**Table 2:** Concentrations of DHN, 1- and 2-NAP, and 1-PYR according to sampling time (IQR: interquartile range, < LOD: < limit of detection).

Sampling		DHN [µg/g creatinine]	1-NAP [µg/g creatinine]	2-NAP [µg/g creatinine]	1-PYR [µg/g creatinine]
1	Median	2.23	0.61	2.00	0.06
(n = 30)	IQR	1.43–4.18	0.27–1.11	1.18–2.88	0.04–0.08
	Range	0.87–10.19	< LOD—4.08	0.30–10.02	0.03–0.15
2	Median	3.32	0.39	1.94	0.05
(n = 28)	IQR	2.03–4.80	0.16–0.77	1.13–3.47	0.03–0.06
	Range	1.41–9.04	< LOD—2.61	0.21–10.94	< LOD—0.22
3	Median	31.60	4.34	7.42	0.06
(n = 26)	IQR	25.79–52.81	2.75–6.17	4.55–9.78	0.04–0.08
	Range	10.68–166.80	0.75–73.88	2.07–42.68	< LOD—0.18
4	Median	134.15	12.08	11.96	0.20
(n = 25)	IQR	92.94–164.89	9.46–15.79	11.05–18.20	0.14–0.31
	Range	43.38–380.43	2.72–74.92	3.97–58.89	0.06–1.61
5	Median	131.46	10.79	10.07	0.40
(n = 22)	IQR	104.83–207.75	6.34–14.35	8.14–14.40	0.21–0.59
	Range	78.66–375.77	2.43–39.36	5.62–23.16	0.16–1.88
6	Median	86.01	4.53	5.12	0.29
(n = 23)	IQR	67.60–137.94	3.95–8.44	3.62–7.98	0.19–0.42
	Range	46.21–284.21	1.73–19.04	1.17–12.78	0.10–1.93
7	Median	51.58	3.33	3.48	0.25
(n = 27)	IQR	40.81–76.67	1.99–5.22	2.34–5.27	0.16–0.30
	Range	21.95–159.57	1.08–12.63	1.30–11.32	0.08–1.56
8	Median	46.02	2.86	3.14	0.21
(n = 27)	IQR	30.70–63.89	1.93–5.59	1.76–4.19	0.12–0.33
	Range	19.82–154.82	0.35–10.73	1.08–10.02	0.06–1.06
9	Median	27.08	2.66	3.26	0.17
(n = 29)	IQR	20.03–40.28	1.51–4.59	1.93–4.24	0.10–0.25
	Range	15.69–139.47	0.48–9.65	1.02–14.56	0.05–0.63

As described elsewhere^6^, the excretion of both naphthols and 1-PYR clearly increased after training, hitting a peak 1 h (1- and 2-NAP) or 3 h (1-PYR) after the end of the training session. As with the naphthols, the excretion of DHN also increased, starting immediately after the exposure and reaching a peak 1 h after the end of the training session. A median peak concentration of 134.2 µg/g crea. was calculated for DHN (sampling 4) while the individual maximum concentration was 380.4 µg/g crea.. Against this, the median concentrations of 1- and 2-NAP (12.08 and 11.96 µg/g crea.) and 1-PYR (0.40 µg/g crea., sampling 5) were substantially lower during peak excretion.

The increase in concentration from before to 1 or 3 h after exposure (peak excretion) was not only evident on a group basis, but rather in all individual sample sets for each of the four biomarkers. However, the increase varied depending on the marker considered. While for 2-NAP and 1-PYR the median increase from baseline (sample 2) to peak excretion (sample 5 for 1-PYR, sample 4 otherwise) was 6.6-fold and 7.9-fold respectively, this effect was much more pronounced for 1-NAP and DHN, whose concentrations increased 36-fold and 41-fold respectively. (see Supplementary Table S1 online).

When comparing the metabolite concentrations in both morning urine samples collected either before (sampling 1) or after exposure (sampling 9), elevated metabolite concentrations were found the morning after exposure. The median increase in post exposure samples was 12.9 fold for DHN, 3.9 fold (1-NAP), 2.8 fold (1-PYR) and 1.5 fold (2-NAP), respectively (Supplementary Table S1).

**Figure 1:**
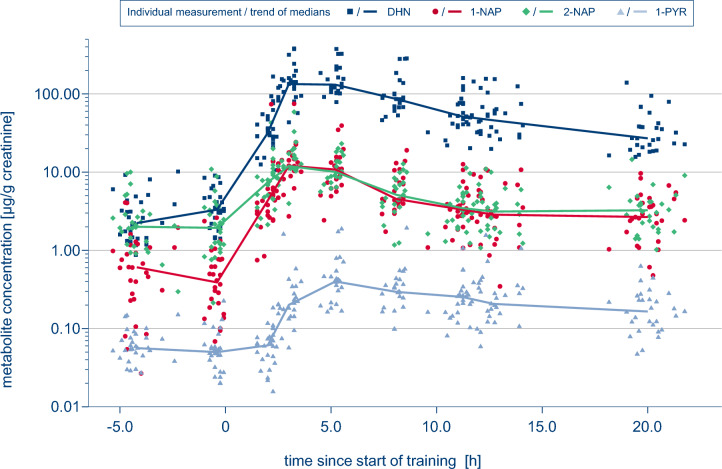
Illustration of the concentration curves for DHN (dark blue squares), 1-NAP (red dots), 2-NAP (green diamonds) and 1-PYR (light blue triangles) with lines marking the time course of the median values calculated for the individual sampling times. Concentration axis in log scale.

The individual values for all markers and sampling times are plotted in Fig. 1: To illustrate the time course of the marker concentrations, lines representing median values for each marker (see also Table 2) and sampling time as specified in Table 1 were added to the graph. Besides the differing concentration levels of the markers pre and post exposure, the high sensitivity of DHN and 1-NAP but not 2-NAP in indicating an additional naphthalene uptake becomes obvious. Moreover, a rather delayed response of 1-PYR after exposure compared to the NAP metabolites as well as the substantially increased metabolite levels for DHN, 1-NAP and 1-PYR 20 h post exposure can be recognised from the graph.

**Figure 2:**
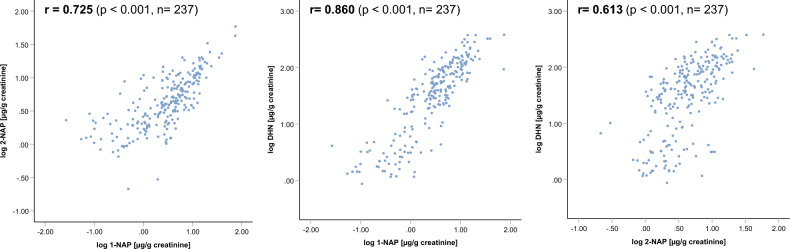
Scatter plots for the bivariate correlations between the logarithmic concentrations of the three naphthalene metabolites 1-NAP. 2-NAP and DHN (r: pearson correlations coefficient, p: significance level).

With regard to the three naphthalene metabolites, correlation analyses revealed significant (*p* < 0.001) associations between the logarithmic concentrations of 1-NAP, 2-NAP and DHN, respectively (Fig. 2). Pearson correlation coefficients of 0.725 (1-NAP – 2-NAP), 0.860 (1-NAP – DHN) and 0.613 (2-NAP –DHN) were calculated for the three associations.

The median concentrations measured at the particular sampling times indicated variable proportions of the individual compounds in the total excretion of the three naphthalene metabolites over time. While the proportion for 1-NAP varied between 4.7 and 12.5% over the whole sampling period, the proportion for 2-NAP dropped from 34 and 41% before exposure to proportions consistently lower than 10% and very similar to the ones of 1-NAP after sampling 3. In contrast, the proportion of DHN increased from 46 and 59% before exposure to values between 80 and 90% for the rest of the sampling period (see Supplementary Fig. S1 online).

After a median time of 3.3 h (range: 2.5–19.0) to reach peak concentrations, an average elimination half-life of 6.6 h (CI 5.4–8.6) was determined for DHN, which is shown with the respective data of 1-NAP, 2-NAP and 1-PYR in Table 3.

**Table 3:** Time to peak and estimated elimination half-lives after peak excretion for the individual markers.

Parameter	Time to peak, median (h)	Time to peak, range (h)	Estimated half-life, mean (h)	Estimated half-life, 95% CI (h)
DHN	3.3	2.5–19.0	6.6	5.4–8.6
1-NAP	3.3	2.2–11.2	6.2	5.1–7.8
2-NAP	3.3	2.2–11.2	5.2	4.4–6.3
1-PYR	5.3	2.5–13.9	7.7	5.9–11.1

## Discussion

In the previous publication on this biomonitoring study^6^, it was already discussed that live fire training is a relevant source of internal PAH exposure for firefighting instructors which is recognizable by significant changes in urinary excretion of monohydroxy PAH metabolites. Of the urinary biomarkers considered, the sum of 1- and 2-NAP showed the strongest increase after time-defined smoke exposure in the previous assessment^6^. In the present further evaluation, we analysed the urinary concentrations of DHN as the main metabolite of NAP. We compared these concentrations to those of 1-NAP and 2-NAP, but also to concentrations of 1-PYR as the most frequently used biomarker of PAH exposure.

The pre-exposure concentrations found for 1-NAP, 2-NAP and 1-PYR presented here correspond to background exposure^6^ with only slight differences between both pre exposure samplings. The highest pre-exposure concentration for the sum of 1- and 2-NAP was 27.48 µg/l (data not shown) and therefore still below the German Biological Reference Value (BAR) of 35 µg/l indicating the upper boundary of environmental background exposure in the general population (non-smokers at working age^22^). Similarly, 1-PYR excretion pre exposure (maximum 0.22. µg/g creatinine, Table 2) did not exceed a concentration of 0.30 µg/g creatinine, which is considered as reference level of internal pyrene exposure in the non-smoking German general population^36,37^. ^36,37^DHN and 2-NAP showed higher baseline concentrations than 1-NAP. For 2-NAP in particular, this is a common finding in the literature and is consistent, for example, with the studies of (Burkhardt et al. 2022). Furthermore, elevated 2-NAP concentrations have been also found in large surveys on PAH background exposure of non-smokers in the US (NAHNES) or Canada (CHMS), as well as in other studies^38–41^.

Based on what is known about the metabolism of naphthalene in mammals, 1-NAP is the preferentially formed and excreted metabolite of the two naphthols, whereas DHN is described as the major metabolite of NAP^42–44^. Therefore, the proportion of 2-NAP among the excreted metabolites should be lower than that of 1-NAP and also DHN after uptake of NAP alone. The observed contrary results in background exposure studies (as mentioned above) suggest the presence of an external source other than NAP for 2-NAP excretion^45^. Possible sources include fragrance compounds such as 2-methoxy- or 2-ethoxynaphthalene or sudan dyes such as 1-phenyl-azo-2-naphthol (Sudan I, solvent yellow 14). All of these carry a 2-NAP moiety which could, at least theoretically, be released by metabolism^46–48^.

Thus, for reasons of specificity, the use of 2-NAP as an indicator of naphthalene uptake appears to be limited at least in the range of environmental background exposure.

DHN was present in all samples. In accordance with the results for the other metabolites, it can be assumed that the measured DHN pre-exposure concentrations also represent general background excretion for this biomarker. The measured values are consistent with literature data, although data on background levels of DHN in urine are rare. Klotz et al. found a median (range) urinary DHN level of 4.6 µg/l (< LOD-19.3 µg/l) in 20 non-smoking subjects^43^. In the control group (n = 22 office and hospital workers including three smokers) for the study of DHN in urine samples from Chinese steelworkers, Wu et al. found a geometric mean (geometric standard deviation) of 38.8 µg/l (2.31 µg/l)^24^. In the 58 urine samples collected in the present study before the onset of exposure to fire emissions, DHN concentrations were in a comparable range of 0.6–19.4 µg/l (median 2.89 µg/l).

There was a measurable concentration increase for all metabolites considered after exposure to fire emissions^6^. During peak excretion of naphthols (sampling 4), 64% of the samples showed concentrations exceeding background exposure of non-smokers (35 µg/l sum of 1- and 2-NAP^49^). In sampling 5, 73% of the samples exceeded the respective reference value for 1-PYR (0.30 µg/g crea^36,37^. However, an upper margin of internal exposure typically found in smokers (e.g. 95th percentiles of 32.8 µg/g crea., 29.0 µg/g crea., and 0.80 µg/g creatinine for 1-NAP, 2-NAP and 1-PYR^41^) was exceeded only in a clear minority (< 14%) of all peak excretion samples^6^. Unfortunately, data on DHN excretion in smokers is scarce. According to Klotz et al.^43^, a trend towards higher concentrations (range 1.9–62 µg/l, median 17.1 µg/l, n = 9 smokers) can be assumed. However, in view of the small study group, the upper border of the range cannot be reliably assessed currently.

The additional uptake of naphthalene becomes most evident by DHN, which is clearly influenced by exposure to fire emissions. The maximum DHN concentrations found in firefighters (median sampling 4: 246.7 µg/l) were higher than the concentrations measured in post shift samples of wood impregnators handling hot creosote (Table 4). Taking into account the concentrations at the end of the shift, corresponding to 71.3 µg/l (16.6–673.6 µg/l) at sampling 6 in our study, the values are in a comparable range. Thus according to DHN, the short-term exposure to fire emissions under respiratory protection in our study seems to result in similar naphthalene body burdens as measured for creosote workers after an 8 h-workshift. Nevertheless, the DHN concentrations of the firefighters are still far below the range defined by the recently published exposure equivalents for carcinogenic substances (EKA correlation: 4.000–34.200 µg/l^50^), describing the association between respiratory naphthalene uptake and renal DHN excretion in workers. Higher urinary DHN concentration have been found for example in in coke oven workers or in workers producing abrasives or maintaining converters (Table 4). Thus, DHN excretion of firefighters in our study indicates a rather low to moderate exposure to NAP from an occupational health perspective.

**Table 4:** Urinary Peak DHN concentrations following live fire training in this study compared with DHN concentrations in male adults exposed to PAHs in other occupations. (GM: geometric mean; GSD: geometric standard deviation).

Study group (n, country)	Statistical measure	Concentration [µg/l]	References
Firefighters after life fire training (n = 6, Germany)	Median (Range)	246.7 (18.2–1084.2)	This study
Coke oven workers (n = 28, China)	GM (GSD)	452 (2.28)	^24^
Workers at converter infeed (n = 26, Germany)	Median (Range)	945.4 (22.0–1902.6)	^43^
Wood impregnation workers (n = 9, Germany)	Median (Range)	88.3 (10.8–1497)	^51^
Workers in the abrasive industry, post-shift Monday (n = 10, Germany)	Median (Range)	3944 (114–42,066)	^25^
Workers in the abrasive industry, post-shift Thursday (n = 10, Germany)	Median (Range)	11,162 (337–51,809)	^25^

The increase in concentrations of 1-NAP and DHN from before exposure up to the maximum of excretion after exposure was comparable with factors of 40 and 35, respectively. Considering all samples, a strong correlation between both markers was found.

In contrast, the concentration of 2-NAP increased only by a factor of about 6 and finally reached maximum levels similar to that of 1-NAP. On the one hand, this can be explained by a higher basal excretion of 2-NAP already before smoke exposure. On the other hand, 2-NAP is metabolically formed to a lesser extent under NAP exposure than 1-NAP and DHN. As a consequence, the correlations including 2-NAP are weaker than the association between DHN and 1-NAP.

Overall, we found variable proportions of the three naphthalene metabolites over time. The initial proportion of DHN of less than 60% changed to proportions up to 90% after exposure. Concurrently, the 2-NAP proportion decreased from a maximum of 41% to less than 10%. After exposure, the proportions remained largely unchanged until the end of the sampling period while the absolute concentrations decreased significantly. A trend towards increasing proportions of DHN within the NAP metabolites with increasing exposure was already described^51^, though not within the same subjects in different exposure states like in our study. In this respect, our findings support the assumptions of Klotz et al., according to which “the formation of mono- and dihydroxylated metabolites in humans depends on the level of naphthalene exposure or of the cumulative PAH exposure”^51^. To add more information on this topic, the further time course of metabolite elimination in exposed firefighters should be studied until pre-exposure concentrations and presumably pre-exposure ratios are regained. Unfortunately, the observation period in our study was too short to re-establish this exposure status.

In the present study, 1-PYR was also evaluated as this biomarker is often measured to assess the PAH exposure of firefighters. Indicating internal exposure within a typical range for firefighting instructors exposed to smoke from treated wood^6^, the 1-PYR concentration showed a lower increase over time than DHN or 1-NAP. Reasons for this may be lower external exposure i.e. considerably lower concentrations of the parent compound PYR in fire emissions when compared to NAP, paired with higher efficacy of protective clothing against less volatile compounds like PYR, which tend to be adsorbed by particles^11,12^. Differing skin exposure, differing kinetics in dermal uptake of NAP and PYR and differing kinetics in elimination as their corresponding metabolites may be also responsible for weaker associations between 1-PYR and the NAP metabolites (correlation coefficients ranging from 0.425 to 0.699, *p* < 0.001, n = 237; data not shown) when compared to the interrelationships between the three NAP metabolites.

The present study of metabolite excretion over time after short-term exposure highlights the importance of the sampling time for biomonitoring. It could be shown that the concentrations of 1- NAP, 2- NAP and DHN reached a maximum 3.3 h after exposure. DHN is subsequently eliminated with a calculated initial half-life of 6.6 h (CI 5.4–8.6). To our knowledge, comparable data are not yet available for this substance. The results on the kinetics of DHN are very similar to those of 1-NAP and 2-NAP. Thus, they clearly differ from the results for 1-PYR, which shows an estimated time to peak of 5.3 h. Internal exposure is therefore indicated by DHN and the other NAP metabolites at an earlier stage than it is by 1-PYR. Thus, the time of sampling for biomonitoring in a setting with short-term exposure to PAHs has to be chosen with respect to the metabolite to be investigated.

No matter which biomarker is regarded, a complete elimination of NAP or PYR absorbed during smoke exposure was not reached within the post exposure observation period of about 18 h. Calculated elimination kinetics for DHN show that, starting from the median maximum exposure (134.15 µg/g crea.), about 5 half-lives or approximately 33 h are required to reach pre exposure levels again, when assuming a monophasic elimination without any additional elimination phase. However, as a biphasic elimination with a slower second elimination phase has to be considered^52^, the estimated time should be a minimum to be adhered to. Overall, this suggests some cumulative effect if the intervals between individual short-term exposures to fire emissions are too short^7^. Accumulation effects may alter the evaluation of the internal exposure as found in this study, which may be rather considered as low to moderate from an occupational health perspective. In case of live fire training units, which can be scheduled such as those carried out in this study, re-exposure should therefore take place at the earliest on day 2 after pre-exposure.

## Concluding remarks

PAHs, and in particular NAP, are relevant components of fire emissions. PAH uptake by firefighting instructors was demonstrated during short-term exposure even under full protection and good hygienic conditions. With regard to NAP biomonitoring, our results show that the urinary metabolites 1-NAP and DHN are particularly suitable as biomarkers, with DHN occurring in higher concentrations. In particular, DHN, which becomes more important as an excretion product with increasing exposure compared to 1-NAP and 2-NAP, was found to be sensitive in assessing the additional uptake of NAP at work. Unlike for 2-NAP, sources other than NAP seem to play a minor role for DHN formation, suggesting also a high selectivity of the marker. The ideal sampling time for recording the maximum internal load after short-term exposure is 3–4 h after the end of exposure. Given the potential of the biomarker, appropriate reference and limit values would be desirable. In addition to the common 1-PYR biomonitoring, our DHN measurements have shown that the protective clothing currently in use provides only limited protection especially against volatile substances. To reduce internal exposure, especially for instructors in training situations, the choice of fuel used (e.g. chipboard vs. untreated wood) could be a starting point^7^. Nevertheless, organisational measures should be taken to avoid daily exposure to smoke in order to prevent cumulative effects. In general, the absorption of volatile substances from the vapor phase through the skin must be regarded. In order to reduce the penetration of volatile substances onto the skin surface, revisions of protective clothing concepts should be considered.

### Supplementary Information

Supplementary Information.

## Data Availability

The datasets used and/or analysed during the current study are available from the corresponding author.
