# Supplementary material for: Renal excretion of 1,2-dihydroxynaphthalene (DHN) in firefighting instructors after exposure to polycyclic aromatic hydrocarbons (PAHs) during live fire training
Source: Sci Rep. 2024 Jul 2;14:15230. doi: 10.1038/s41598-024-62388-2 (PMC11219744; doi:10.1038/s41598-024-62388-2)
Supplement: Supplementary file 1 — Supplementary Information. [file 41598_2024_62388_MOESM1_ESM.docx]

# Supplementary information

Renal excretion of 1,2-dihydroxynaphthalene (DHN) in firefighting instructors after exposure to polycyclic aromatic hydrocarbons (PAHs) during live fire training.

Felix Lang^1*^, Daniel Wollschläger^2^, Stephan Letzel^1^, Bernd Roßbach^1^

1) Institute of Occupational, Social and Environmental Medicine, University Medical Center, Johannes Gutenberg-University, Mainz, Germany

2) Institute of Medical Biostatistics, Epidemiology and Informatics (IMBEI), University Medical Center, Johannes Gutenberg-University, Mainz, Germany

*Corresponding Author: felilang@uni-mainz.de

Table S 1: Ratio of concentrations immediately before exposure (sampling 2) to peak excretion (DHN, 1- and 2-NAP: sampling 4, 1-PYR: sampling 5) and ratio of concentrations in the morning before exposure (sampling 1) and the morning after exposure (sampling 9). Median and ranges calculated from ratios on personal level.

| Parameter | Ratio of concentrations sampling 2 vs.  peak excretion | Ratio of concentrations sampling 1 vs. sampling 9 |
| --- | --- | --- |
|  | **Median**  (range) | **Median**  (range) |
| DHN | **41.1** | **12.9** |
|  | (13.6 - 165.5) | (2.3 - 79.7) |
| 1-NAP | **36.3** | **3.9** |
|  | (3.9 - 330.8) | (1.1 - 43.9) |
| 2-NAP | **6.6** | **1.5** |
|  | (1.8 - 275.5) | (0.6 - 6.7) |
| 1-PYR | **7.9** | **2.8** |
|  | (1.9 - 38.6) | (1.0 - 10.4) |

Figure S 1: Proportions of the individual naphthalene metabolites among all naphthalene metabolites at the respective sampling times (calculation based on median concentrations).
